# Circulation of avian paramyxoviruses in wild birds of Kazakhstan in 2002–2013

**DOI:** 10.1186/s12985-016-0476-8

**Published:** 2016-02-04

**Authors:** Kobey Karamendin, Aidyn Kydyrmanov, Aigerim Seidalina, Saule Asanova, Klara Daulbayeva, Yermukhammet Kasymbekov, Elizaveta Khan, Sasan Fereidouni, Elke Starick, Kainar Zhumatov, Marat Sayatov

**Affiliations:** Institute of Microbiology and Virology, 103 Bogenbay batyr Str, Almaty, 050010 Kazakhstan; Friedrich Loeffler Institute, Greifswald, Insel Riems Germany; WESCA Wildlife Network, Greifswald, Germany; Present Address: University of Veterinary Medicine Vienna, Research Institute of Wildlife Ecology, Vienna, Austria; Kazakh National Agrarian University, 8 Abay Str., 050010, Almaty, Kazakhstan

**Keywords:** Avian paramyxovirus, Kazakhstan, Wild bird, Sequencing, Monitoring, Phylogenetics

## Abstract

**Background:**

Screening wild birds for avian paramyxoviruses is of increasing importance. 6913 samples of tracheal and cloacal swabs were collected during 2002–2013 and tested to study the prevalence of APMVs in wild avifauna of Kazakhstan. As a result, 45 isolates were obtained during this period and their ecological niches and genetic relationships were defined.

**Methods:**

Tracheal and cloacal samples from wild birds were collected using sterile swabs placed in viral transport medium and kept in liquid nitrogen until delivery to the laboratory. Samples were inoculated into 10-day-old embryonated chicken eggs and reverse transcription PCR (RT-PCR) assays were performed via a one-step protocol. The PCR products were sequenced and phylogenetic trees were constructed using the ‘Neighbour Joining’ method.

**Results:**

Six thousand nine hundred thirteen samples from 183 bird species were investigated and 45 isolates belonging to four different serotypes APMV-1, APMV-4, APMV-6 and APMV-8 were identified. All APMVs were isolated predominantly from birds belonging to *Anatidae* family (ducks and geese) and only one APMV-4 isolate was obtained from shorebird (Curlew) on the Caspian seashore. Genetic studies showed that the recovered APMV-1 strains had highest homology with European isolates. APMV-4 strains isolated in 2003, and APMV-6 and APMV-8 isolated in 2013 were 99 % identical to isolates from Far East.

**Conclusion:**

This is the first reported characterization of avian paramyxoviruses from wild birds isolated in Kazakhstan. These data confirm the wide distribution of APMV-1, APMV-4 and APMV-6 in the Asian subcontinent. The obtained data contribute to the accumulation of knowledge on the genetic diversity and prevalence of APMVs in wild bird populations.

**Electronic supplementary material:**

The online version of this article (doi:10.1186/s12985-016-0476-8) contains supplementary material, which is available to authorized users.

## Background

Paramyxoviruses (PMV) are negative-strand RNA viruses circulating among mammals, birds and reptiles. Their viral RNA encodes six major proteins: nucleocapsid protein (NP); phosphoprotein (P); matrix protein (M); fusion protein (F); haemagglutinin-neuraminidase (HN) and large RNA polymerase (L) as well as two non-structural proteins V and W [1]. The APMV-6 serotype has a unique additional protein SH which is not found in the other serotypes. Its biological function in APMVs is not known, but it is hypothesized to play an important role in blocking the TNF-α-mediated apoptosis pathway in other *Paramyxoviridae* representatives including SV5 and Mumps virus [2].

Avian paramyxoviruses (APMV) belong to the genus Avulavirus of Paramyxoviridae family and to date has been found to cause diseases with varying clinical manifestations in more than 200 wild and domestic bird species. They are divided into twelve serotypes based on a haemagglutination inhibition assay (APMV-1 –12) [3].

The most dangerous for poultry is APMV-1 (Newcastle disease virus), which is widespread in poultry around the world and inflicts significant economic damage. The disease ranges from asymptomatic infection to severe epizootics, often resulting in complete loss of commercial flocks.

Little is known about the molecular and biological characteristics and pathogenicity of other APMV serotypes. In poultry they primarily cause respiratory infection or intestinal disease with varying degree of pathogenicity accompanied by a decrease in egg production, weight gain, conjunctivitis and pneumonia with variable mortality rates [4]. APMV-4 and 8 have been isolated from ducks and other wild birds with no clinical signs of disease [5, 6]. APMV-6 virus causes mild respiratory disease and is associated with a drop in egg production in turkeys [7]. Experimental infection of chickens with APMV-4 and APMV-6 showed mild interstitial pneumonia, catarrhal tracheitis, and BALT or GALT hyperplasia, suggestive of viral disease [8]. APMV-8 is a rare serotype that was first isolated in 1976 from a feral Canadian goose in the United States of America. APMV-10-12 have been isolated exclusively from wild birds with no evidence of any disease.

In Kazakhstan APMV-1 was first isolated in 1982 from synanthropic birds (house and tree sparrow, grey crow, magpie). In February 2005 it was isolated from wild pigeons during a mass-mortality [9, 10]. Other serotypes of APMV have not previously been isolated and described in Kazakhstan before this research aside from APMV-2 strains from domestic poultry in 1987–1989 [11].

**Fig. 1:**
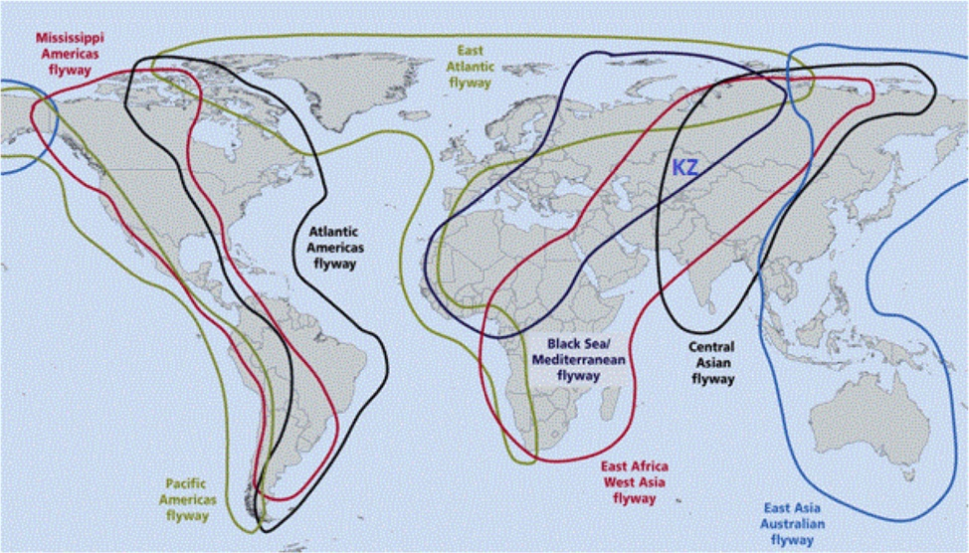
Major Flyways crossing Kazakhstan (KZ) © www.eaaflyway.net/about/the-flyway/

Kazakhstan has a vast territory crossed by intercontinental flyways, and hundreds of bird species concentrate in wetlands during migration and breeding periods (Fig. 1). Here we present the results of the first large-scale ecological study of APMV circulation among wild birds in the Central Asian region, isolation of the contemporary virus variants and definition of their ecological niches and genetic relationships.

## Results

### Virus detection and identification

Large scale monitoring of wild birds, focused mainly on AIV and APMV, was conducted in 2002–2013 and resulted in 6913 samples collected from 17 avian orders, 38 families and 183 species. Taxa from a range of niches were sampled, including aquatic (84 %), and terrestrial (16 %) avifauna. (Additional file 1: Table S1).

**Fig. 2:**
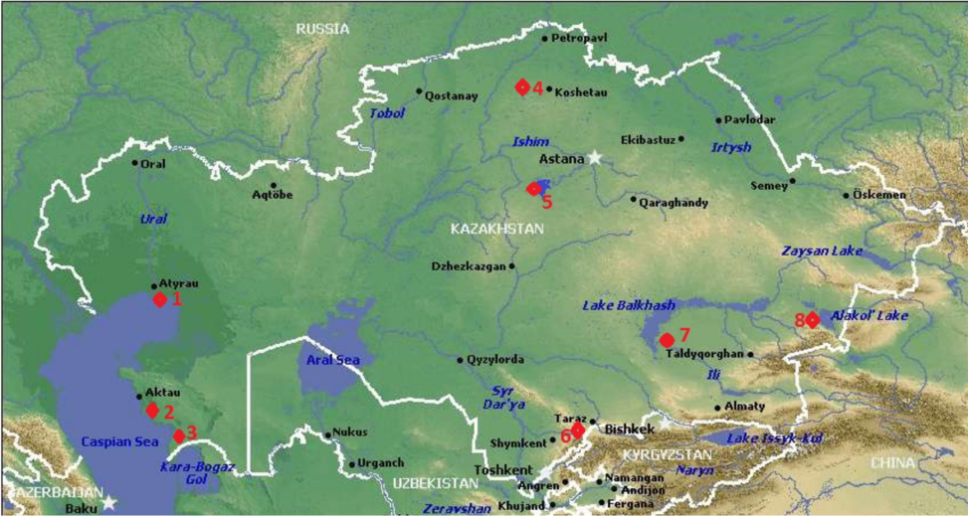
Places of sampling in Kazakhstan. © www.kazakhstandiscovery.com/kazakhstan-map.html. Sampling locations in Kazakhstan are marked with red rhombs. Numeration of sampling sites are the following: 1-Northern Caspian, 2 and 3 – Middle Caspian (Western Kazakhstan); 4 – Northern Kazakhstan; 5 – Central Kazakhstan; 6 – Southern Kazakhstan; 7 – Balkhash Lake, 8 – Alakol Lake (Southeastern Kazakhstan)

Sampling sites covered almost all migrating birds’ nesting and resting sites: Tengiz-Korgalzhyn Lakes System – Central and Northern Kazakhstan, Caspian seashore – Western Kazakhstan, Balkhash-Alakol lakes system in South-Eastern Kazakhstan, and Chokpak ornithological station in Southern Kazakhstan (Fig. 2).

Overall, 45 APMV strains were isolated following culture in embryonated chicken eggs. After sequencing of PCR products and subsequent BLAST analysis it was determined that 23 isolates belonged to APMV-1, thirteen isolates to APMV-4, one isolate to APMV-6 and eight isolates to APMV-8 (Table 1). Out of 6913 samples, 45 were positive and the isolation rate was 0.65 %.

**Table 1:** Avian species samples positive for APMVs in Kazakhstan, 2002-2013

Order	Family	Species	No of samples	No of positive			
				APMV-1	APMV-4	APMV-6	APMV-8
*Anseriformes*	*Anatidae*	Whooper Swan	7				1
		Greylag Goose	175	5			
		White-fronted Goose	173	2			4
		Bean	7				2
		Lesser white-fronted Goose	27				
		Ruddy Shelduck	334	4			
		Mallard	291	3	1		
		Gadwall	145		5		
		Wigeon	45	4			
		Pintail	103	1			
		Shoveler	41		3		
		Teal	191	2	3		
		Common pochard	88	1			
		Red-crested Pochard	234	1		1	
*Charadriiformes*	*Scolopacidae*	Curlew	5		1		
		Little stint	169				1
Total				23	13	1	8

APMV-1 was isolated for the first time in Central Kazakhstan in 2005 in graylag geese and later in the same region in 2006 and 2009. In 2008 and 2013 other viruses of this serotype were isolated from ducks in Southern Kazakhstan. All viruses were identified in birds belonging to *Anatidae* family (ducks and geese), 7 isolates from geese and 16 from ducks of various species.

APMV-4 was isolated in 2003 near the Alakol lake close to the Chinese border. The second isolation of this serotype was made in the Northern Caspian in 2009. Viruses of this serotype were also isolated predominantly from *Anatidae* family but only one was obtained from a shorebird (Curlew) on Caspian seashore. APMV-6 was isolated once from a duck in Alakol Lake in Southeastern Kazakhstan in 2013. APMV-8 was isolated twice in 2013 in samples from two separate geographically distant regions of Northern Caspian and Southern Siberia (Table 2).

**Table 2:** Spatial and temporal characterization of APMV-positive samples collected in Kazakhstan in 2002-2013

Serotype	No of isolates	Region	Year/ Collected Samples					
			2003/158	2005/662	2006/586	2008/1088	2009/1392	2013/486
APMV-1	*23*	Central Kazakhstan		1	16		3	
		Southern Kazakhstan				2		1
APMV-4	*13*	South-eastern Kazakhstan	1					
		Western Kazakhstan					12	
APMV-6	*1*	South-eastern Kazakhstan						1
APMV-8	*8*	Northern Kazakhstan						7
		Southern Kazakhstan						1
Total			1	1	16	2	15	10

The prevalence of APMV positive infection varies each year between 0.15 % in 2005 and 2.73 % in 2006. The mean prevalence over a 12 year period was 0.65 %.

The most prevalent serotypes were APMV-1 (0.33 % all years and 2.73 % in 2006), APMV-4 (0.18 % and 0.86 % in 2009), APMV-8 (0.11 % and 1.64 in 2013) and APMV-6 (0.01 % and 0.63 % in 2003), respectively.

Among various sampling locations, the APMVs were detected most frequently in Central Kazakhstan (44 %, all APMV-1), Western Kazakhstan (26 %, all APMV-4), Northern Kazakhstan (15 %, all APMV-8), Southern Kazakhstan ( 9 %, APMV-1 and APMV-8) and South-eastern Kazakhstan (5 %, APMV-4 and APMV-6), respectively.

### Genetic characterization

Representatives of each APMV serotype strain from all various times and regions were selected for sequencing. The F genes of APMV-1, 4 and the complete genomes of APMV-6 and 8 were sequenced.

BLAST search of the novel APMV-1 strains showed the highest homology (99 %) with a European isolate APMV-1/Pochard/Finland/13193/06. APMV-4 strains isolated in 2009 were 99 % homologous to European isolate APMV4/Teal/Djankoy/9-17-11/10 and one APMV-4 strain isolated in 2003 was 99 % identical to Korean isolate APMV-4/KR/YJ/2006.

APMV-6 isolate showed the highest (99 %) homology with a Chinese isolate mallard/Jilin/190/2011 and APMV-8 isolates were 99 % homologous to Japanese strain pintail/Wakuya/20/78.

Analysis of the deduced amino acid motifs for the fusion protein cleavage site of isolated APMVs showed that all of them are of an avirulent type containing one or two basic amino acids (Table 3). Velogenic APMV-1 strains contain a multibasic cleavage site having consensus sequence of RRQKRQF with a polybasic furin motif RX[R/K]RQ that is cleaved by intracellular furin or furin-like protease. Lentogenic APMV-1 strains lack this polybasic site and need extracellular trypsin like protease found in the epithelium of the respiratory and intestinal tracts [12].

**Table 3:** Analysis of the deduced amino acid motifs for the fusion protein cleavage site

APMV-1	
APMV-1/greylag goose/Astana/1375/2005	SGGERQERLVG
APMV-1/pintail/Korgalzhyn/1786/2006	SGGERQERLVG
APMV-1/wigeon/Korgalzhyn/1819/2006	SGGERQERLVG
APMV-1/red crested pochard/Korgalzhyn/3645/2009	SGGERQERLVG
APMV-1 /slender-billed gull/Korgalzhyn/3651/2009	SGGERQERLVG
APMV-1/ shelduck/Chokpak/5717/2013	SGGERQERLVG
APMV-1/chicken/Almaty/41/2013	SGGRRQKRFIG
APMV-4	
APMV-4/Gadwall/Aktau/3825/09	RDADIQPRFIG
APMV-4/Shoveler/Aktau/3819/09	RDADIQPRFIG
APMV-4/Curlew/Aktau/3818/09	RDADIQPRFIG
APMV-4/Teal/Aktau/3820/09	RDADIQPRFIG
APMV-4/gadwall/Alakol/386/04	RDADIQPRFIG
APMV −6	
APMV-6/red-crested_pochard/Bakanas/5842/2013	QNPAPEPRLIG
APMV-8	
APMV-8/Little Stint/Chokpak/5669/2013	SETYPQTRLIG
APMV-8/white-fronted goose/Northern Kazakhstan/5747/2013	SETYPQTRLIG
APMV-8/ white-fronted goose/Northern Kazakhstan/5749/2013	SETYPQTRLIG
APMV-8/ Bean Goose/Northern Kazakhstan/ 5764/2013	SETYPQTRLIG
APMV-8/white-fronted goose/Northern Kazakhstan/5765/2013	SETYPQTRLIG

During evaluation of in vitro replication of APMVs it was determined that of APMV 2–9 serotypes only APMV-2, −3 and −5 replicate efficiently in cell culture, but only two latter serotypes induced syncytium formation in infected cells [4]. The other APMVs (serotypes 4, 6, 7, 8, and 9) have one or two basic amino acids in their cleavage site sequences and exhibited single cell infection and inefficient replication in vitro.

Phylogenetic analysis of the F- gene of Kazakhstan APMV-1 strains was conducted to define their relationships with other viruses from GenBank. Kazakhstan APMV-1 strains from wild birds have 99 % similarity with Finnish isolate and so they formed a monophyletic group inside the class 1, consisting of non-pathogenic viruses.

**Fig. 3:**
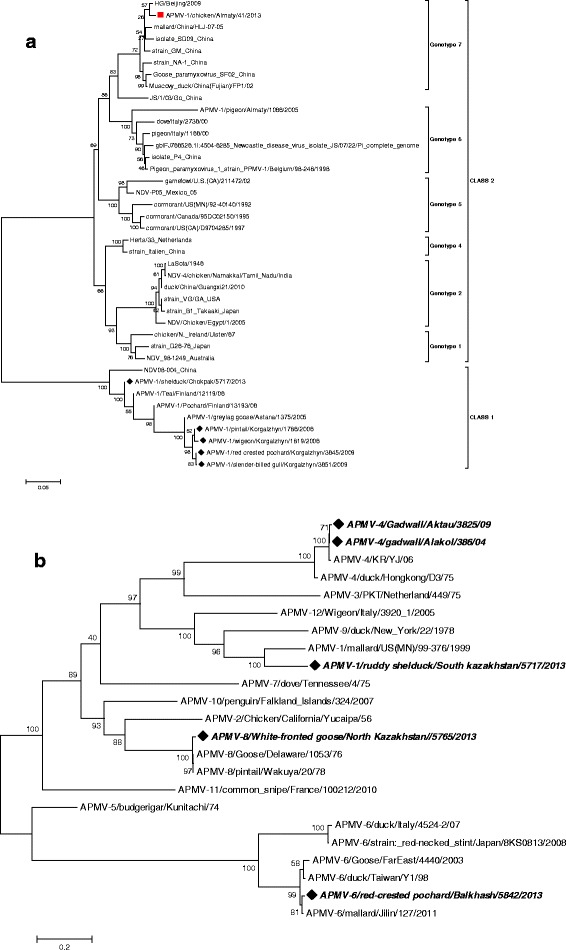
Phylogenetic analyses of the APMV-1 (**a**) and other serotypes (**b**) from Kazakhstan. Isolates from the Kazakhstan wild birds are marked with black diamonds, velogenic APMV-1 strain is marked with red square

The phylogram (Fig. 3a), suggests that the velogenic NDV strain APMV-1/chicken/Almaty/41/2013 (Genotype VII) that caused a NDV outbreak in poultry in Almaty region in 2013, is not closely related to the wild APMV-1 isolates presented here. Combined phylogenetic analysis of the other serotype viruses (Fig. 3b), showed that APMV-6 isolates formed a monophyletic group with Jilin/2011 isolate from China. APMV-8 formed a separate branch from other viruses known to date (Delaware/76 and Wakuya/78).

## Discussion

Central Asia is a very important region where flyways connecting the Far East, Europe, Siberia and Southern Asia cross each other. Particularly important is the Caspian seashore, where cases of reassortment among avian influenza viruses of American and Eurasian lineages have been detected [13].

Kazakhstan has a vast territory where three large flyways of water birds cross: Black Sea-Mediterranean, West Asian-East African and Central Asian. Numerous places of nesting, moulting and summer concentration of birds play an important role in the maintenance of the populations of more than 150 water bird species inhabiting Central Asia.

There is a limited research on circulation of APMVs among wild birds in Kazakhstan and globally. It is known that wild birds serve as the main natural reservoir for APMV serotypes. Among them APMV-1 is the most widespread [14] followed by APMV-4 and 6 [15]. Other serotypes, APMV-2 [16, 17], APMV-3 [18, 19], APMV-7 [16] and APMV-9 [20] appear to be comparatively rare in wild avifauna. Recently, novel strains, APMV-10-12 have been isolated exclusively from wild birds [2, 13, 21, 22]. APMV-1, 4, 6 and 8 have previously been isolated and reported from other countries in Asia [23–26], and we also detected all the above serotypes in wild avifauna of Kazakhstan in this study.

APMV-1 is a globally spread pathogen [1] and its isolation in Kazakhstan during 2003–2013 confirms this assertion. The main potential threat of this serotype is that low pathogenic strains can become highly pathogenic after several passages through poultry [27]. It was revealed that APMV-1 isolated in this study from wild birds are of another origin from strains caused lethal outbreaks in poultry in 2013 in Southeastern Kazakhstan (unpublished). All APMV-1 isolates in this research were isolated from ducks and geese considered to be main carriers of this serotype in Eurasia. In contrast, in the Americas APMV-1 viruses were isolated from shorebirds and gulls [28, 29].

APMV-4 is widely spread in Asia and globally [30–32]. Comparative phylogenetic analysis of the F-gene of the isolate APMV-4/gadwall/Alakol/386/04 showed the greatest similarity to Korean isolate APMV-4/KR/YJ/06 and Chinese isolate APMV4/duck/China/G302/2012. We propose that viruses of this cluster circulate independently in Asia. APMV-4 isolates from 2009 were almost identical to Ukrainian isolate APMV4/Teal/Djankoy/9-17-11/10, suggesting dissemination via flyways shared with European birds. It is suggested that Korean APMV-4 from the Far East and European isolates from Belgium viruses evolved separately from the reference strain APMV-4/duck/HK/D3/75 [24]. All known APMV-4 strains were isolated from ducks and geese, except one Kazakhstan strain obtained from a shorebird.

Genetic studies of the F-gene of APMV-6 has shown divergence into two separate clusters: the first consists of viruses (including a Kazakh strain), ascending to reference strain APMV-6/duck/Hong Kong/18/199/1977; the second is represented by viruses with a deletion of four nucleotides in their genomes, APMV-6/red-necked stint/Japan/8KS0813/2008 and APMV-6/duck/Italy/IT4524-2/2007 [33].

The new Kazakh virus was shown to be close to APMV-6/mallard/Jilin/127/2011 (98.8 %), which can be explained by the common border between Kazakhstan and China, where wild ducks flyways cross.

As of today APMV-8 strains have been isolated only twice – in the USA and Japan in the 1970s. [34, 35]. A APMV-8 serotype was isolated again in Japan in 2007 from a tundra swan [23]. Thus isolation of this serotype in two regions remote from each other in Kazakhstan is a very rare case, which substantially increases our knowledge about its distribution and evolution.

The reported large scale monitoring resulted in isolation of 45 APMV strains belonging to four serotypes APMV-1, 4, 6 and 8 will enhance the understanding the paramyxoviruses circulation in wild birds of Central Asia and the world.

This study confirms the primary role of wild bird populations as a reservoir of APMV-4, 6 and 8 in nature and potential source of genetic material for the emergence of epizootic APMV-1 variants.

## Methods

### Samples collection

Samples from wild birds were collected in three ways: cloacal and tracheal swabs were obtained from birds captured by ornithologists for banding during studies of migratory patterns, sampling during seasonal hunting for wild birds and samples from freshly dropped faeces of wild aquatic birds.

Samples from terrestrial birds were mostly collected in Chokpak ornithological station in Southern Kazakhstan during banding. Samples from aquatic birds were collected in spring and autumn during mass concentrations, and samples from terrestrial birds throughout the year.

Tracheal and cloacal samples from birds were taken using sterile swabs placed in viral transport medium containing 2000 U/ml penicillin, 2 mg/ml streptomycin, 50 μg/ml gentamycin, 50 U/ml nystatin and 0.5 % bovine serum albumin. During the fieldwork, samples were kept in liquid nitrogen, and were stored at −70 °C after return to the laboratory.

### Virus isolation

Samples were firstly screened by RT-PCR targeting the M gene of avian influenza viruses (AIV). Then negative for AIV samples were inoculated into 10-days-old embryonated chicken eggs (ECE) and incubated 48 hours at +36°С [36]. The presence of the virus in allantoic fluid was detected using hemagglutination assay with chicken red blood cells.

### RNA extraction and RT-PCR

Viral RNA was extracted from 140 μl of hemagglutination assay positive allantoic fluid using the QIAamp RNA Mini kit (Qiagen, Hilden, Germany) in accordance with the manufacturer’s recommendations.

Reverse transcription PCR (RT-PCR) assays were performed on the basis of one-step protocol using appropriate RT-PCR kit (AccessQuick One-Step RT-PCR Kit, Promega) according to the manufacturer’s instructions. Pan-PMV primers targeting the conservative fragment of L-gene were used [37]. The cycling conditions consisted of 45 min at 48 ° C (reverse transcription) and then an initial denaturation at 94 ° C for 2 min followed by 35 cycles of 94 ° C for 15 s, 50 ° C for 30 s, and 72 ° C for 30 s and a final extension at 72 ° C for 10 min. The final PCR products were visualized on 2 % agarose gel.

The pan-PMV primers did not amplify the APMV-4 subtype and therefore we used our own degenerate primer pair targeting 1481 bp fragment of the F-gene: APMV-4 Fgene-Fw – CAAAGTCYGARGGGATTAGGG and APMV-4 Fgene Rv – CACCGAGAACAAYAATMAGACC.

### Sequencing and molecular characterization

After purification, the PCR products were sequenced using the same PCR primers on an ABI 3730xl DNA analyser (Applied Biosystems, USA) using BigDye Terminator Kit v.3.1 Sequencing Kit (Applied Biosystems).

For complete genome sequencing, viral RNA was used as a template for double-stranded cDNA synthesis (Roche). A Covaris M220 ultrasonicator was used for DNA fragmentation. For library preparation, Illumina adaptors (Bioo Scientific) and SPRIworks fragment library cartridge II (Beckman Coulter) were used, with manual size selection afterward. The quality of the library was checked on a Bioanalyzer 2100 (Agilent Technologies). Quantity was determined via quantitative PCR (qPCR) with the Kapa library quantification kit (Kapa Biosystems). Paired-end sequencing was performed on an Illumina MiSeq instrument using the MiSeq reagent kit version 3. Raw sequence data were analyzed and assembled using the Genome Sequencer software suite (version 2.8; Roche).

Obtained nucleotide sequences were aligned using Mega 5.0 [38] software and were deposited in the GenBank data base under accession numbers [Genbank:KP762799, KU366512-KU366520]. Fifty one other full-length and partial sequences from public databases were used for this analysis. Phylogenetic trees were constructed by comparing sequences of the isolated APMVs with respective nucleotide sequences from GenBank using the ‘Neighbour Joining’ method and the Tamura-Nei model in MEGA 5.0 software [39], with 1000 bootstrap replications to assign confidence levels to branches.

## Conclusions

6913 samples from 183 wild bird species were collected during 2002-2013 and tested to study the prevalence of APMVs in avifauna of Kazakhstan. As a result, 45 strains belonging to four APMV serotypes were isolated during this period The obtained data confirm the wide distribution of APMV-1, APMV-4 and APMV-6 in the Asian subcontinent and contribute to the accumulation of knowledge on the genetic diversity and prevalence of APMVs in wild bird populations.

## Additional file

Additional file 1: Table S1. Avian species tested for APMVs in Kazakhstan, 2002–2013. (DOCX 48 kb)

## Abbreviations

APMV: avian paramyxoviruses
RNA: ribonucleic acid
AIV: avian influenza virus
RT-PCR: reverse transcription polymerase chain reaction
