# Supplementary material for: Circulation of avian paramyxoviruses in wild birds of Kazakhstan in 2002–2013
Source: Virol J. 2016 Feb 4;13:23. doi: 10.1186/s12985-016-0476-8 (PMC4743248; doi:10.1186/s12985-016-0476-8)
Supplement: Additional file 1: Table S1. — Avian species tested for APMVs in Kazakhstan, 2002–2013. (DOCX 48 kb) [file 12985_2016_476_MOESM1_ESM.docx]

Table S1. Avian species tested for APMVs in Kazakhstan, 2002-2013

| **Order** | **Family** | **Species** | **No of samples** | **No of positive** | | | | |
| --- | --- | --- | --- | --- | --- | --- | --- | --- |
|  |  |  |  | APMV-1 | | APMV-4 | APMV-6 | APMV-8 |
|  |  | Aquatic bird sp. | 5 |  | |  |  |  |
| ***Podicipediformes*** | *Podicipedidae* | Great crested Grebe | 74 |  | |  |  |  |
|  |  | Little Grebe | 3 |  | |  |  |  |
|  |  | Red-necked Grebe | 3 |  | |  |  |  |
|  |  | Black-necked Grebe | 8 |  | |  |  |  |
| ***Pelecaniformes*** | *Pelecanidae* | White Pelican | 12 |  | |  |  |  |
|  |  | Dalmatian Pelican | 46 |  | |  |  |  |
|  | *Phalacrocoracidae* | Cormorant | 116 |  | |  |  |  |
|  |  | Pygmy Cormorant | 9 |  | |  |  |  |
| ***Ciconiformes*** | *Ardeidae* | Egret | 1 |  | |  |  |  |
|  |  | Grey Heron | 104 |  | |  |  |  |
|  |  | Yellow Heron | 2 |  | |  |  |  |
|  |  | Little bittern | 2 |  | |  |  |  |
|  |  | Night Heron | 80 |  | |  |  |  |
| ***Phoenicopteriformes*** | *Phoenicopteridae* | Flamingo | 140 |  | |  |  |  |
| ***Anseriformes*** | *Anatidae* | Mute Swan | 300 |  | |  |  |  |
|  |  | Whooper Swan | 7 |  | |  |  | 1 |
|  |  | Greylag Goose | 175 | 5 | |  |  |  |
|  |  | White-fronted Goose | 173 | 2 | |  |  | 4 |
|  |  | Bean | 7 |  | |  |  | 2 |
|  |  | Lesser white-fronted Goose | 27 |  | |  |  |  |
|  |  | Red-breasted Goose | 4 |  | |  |  |  |
|  |  | Shelduck | 20 |  | |  |  |  |
|  |  | Ruddy Shelduck | 334 | 4 | |  |  |  |
|  |  | Mallard | 291 | 3 | | 1 |  |  |
|  |  | Gadwall | 145 |  | | 5 |  |  |
|  |  | Wigeon | 45 | 4 | |  |  |  |
|  |  | Pintail | 103 | 1 | |  |  |  |
|  |  | Shoveler | 41 |  | | 3 |  |  |
|  |  | Teal | 191 | 2 | | 3 |  |  |
|  |  | Garganey | 74 |  | |  |  |  |
|  |  | Ferruginous duck | 1 |  | |  |  |  |
|  |  | Common pochard | 88 | 1 | |  |  |  |
|  |  | Red-crested Pochard | 234 | 1 | |  | 1 |  |
|  |  | Tufted Duck | 94 |  | |  |  |  |
|  |  | Greater Scaup | 8 |  | |  |  |  |
|  |  | Long-tailed Duck | 2 |  | |  |  |  |
|  |  | Goldey Eye | 6 |  | |  |  |  |
|  |  | Goosander | 2 |  | |  |  |  |
|  |  | Red-breasted Merganser | 4 |  | |  |  |  |
|  |  | Smew | 3 |  | |  |  |  |
| ***Falconiformes*** | *Accipitridae* | Black Kite | 3 |  | |  |  |  |
|  |  | Sparrow-hawk | 28 |  | |  |  |  |
|  |  | Hen harrier | 2 |  | |  |  |  |
|  |  | Pallid Harrier | 6 |  | |  |  |  |
|  |  | Steppe Eagle | 2 |  | |  |  |  |
|  |  | Long-legged Buzzard | 4 |  | |  |  |  |
|  |  | Common Buzzard | 2 |  | |  |  |  |
|  | *Falconidae* | Saker falcon | 2 |  | |  |  |  |
|  |  | Red-footed falcon | 2 |  | |  |  |  |
|  |  | Hobby | 4 |  | |  |  |  |
|  |  | Kestrel | 2 |  | |  |  |  |
| ***Galliformes*** | *Phasianidae* | Chukar | 7 |  | |  |  |  |
|  |  | Grey partridge | 1 |  | |  |  |  |
|  |  | Quail | 18 |  | |  |  |  |
|  |  | Pheasant | 24 |  | |  |  |  |
| ***Gruiformes*** | *Gruidae* | Crane | 4 |  | |  |  |  |
|  | *Rallidae* | Moorhen | 10 |  | |  |  |  |
|  |  | Coot | 308 |  | |  |  |  |
| ***Charadriiformes*** | *Charadriidae* | Little ringed plover | 37 |  | |  |  |  |
|  |  | Kentish plover | 14 |  | |  |  |  |
|  |  | Ringed plover | 5 |  | |  |  |  |
|  |  | Temminck's Stint | 4 |  | |  |  |  |
|  |  | Purple sandpiper | 6 |  | |  |  |  |
|  |  | Lapwing | 26 |  | |  |  |  |
|  | *Recurvirostridae* | Black-winged stilt | 17 |  | |  |  |  |
|  |  | Avocet | 9 |  | |  |  |  |
|  | *Scolopacidae* | Black-tailed godwit | 5 |  | |  |  |  |
|  |  | Blar-tailed godwit | 4 |  | |  |  |  |
|  |  | Snipe | 8 |  | |  |  |  |
|  |  | Grey Phalarope | 4 |  | |  |  |  |
|  |  | Red-necked Phalarope | 22 |  | |  |  |  |
|  |  | Curlew | 5 |  | | 1 |  |  |
|  |  | Jack snipe | 7 |  | |  |  |  |
|  |  | Spotted redshank | 3 |  | |  |  |  |
|  |  | Sandpiper | 2 |  | |  |  |  |
|  |  | Marsh sandpiper | 52 |  | |  |  |  |
|  |  | Redshank | 170 |  | |  |  |  |
|  |  | Broad-billed sandpiper | 6 |  | |  |  |  |
|  |  | Common sandpiper | 22 |  | |  |  |  |
|  |  | Terek sandpiper | 10 |  | |  |  |  |
|  |  | Greenshank | 10 |  | |  |  |  |
|  |  | Grey plover | 7 |  | |  |  |  |
|  |  | Ruff | 252 |  | |  |  |  |
|  |  | Little stint | 169 |  | |  |  | 1 |
|  |  | Curlew sandpiper | 2 |  | |  |  |  |
|  |  | Dunlin | 16 |  | |  |  |  |
|  |  | Turnstone | 10 |  | |  |  |  |
|  |  | Wood sandpiper | 445 |  | |  |  |  |
|  |  | Collared Pratincole | 2 |  | |  |  |  |
|  | *Laridae* | Gull sp. | 6 |  | |  |  |  |
|  |  | Great black-headed gull | 352 |  | |  |  |  |
|  |  | Black-headed gull | 277 |  | |  |  |  |
|  |  | Herring gull | 115 |  | |  |  |  |
|  |  | Common gull | 61 |  | |  |  |  |
|  |  | Yellow-legged gull | 189 |  | |  |  |  |
|  |  | Slender-billed gull | 10 |  | |  |  |  |
|  |  | Little gull | 66 |  | |  |  |  |
|  | *Sternidae* | Caspian tern | 120 |  |  | |  |  |
|  |  | Common tern | 46 |  |  | |  |  |
|  |  | Gull-billed tern | 28 |  |  | |  |  |
|  |  | Sandwich tern | 3 |  |  | |  |  |
|  |  | Little tern | 22 |  |  | |  |  |
|  |  | White-winged black tern | 2 |  |  | |  |  |
|  |  | Black tern | 4 |  |  | |  |  |
| ***Pteroclidifomes*** | *Pteroclididae* | Black-bellied sandgrouse | 2 |  |  | |  |  |
| ***Columbiformes*** | *Columbidae* | Rock dove | 1 |  |  | |  |  |
|  |  | Loughing dove | 26 |  |  | |  |  |
|  |  | Rufous turtle dove | 6 |  |  | |  |  |
|  |  | Stock dove | 22 |  |  | |  |  |
|  |  | Woodpigeon | 2 |  |  | |  |  |
| ***Cuculiformes*** | *Cuculinae* | Cuckoo | 6 |  |  | |  |  |
| ***Strigiformes*** | *Strigidae* | Short-eared owl | 2 |  |  | |  |  |
|  |  | Scops owl | 6 |  |  | |  |  |
|  |  | Long-eared owl | 1 |  |  | |  |  |
| ***Caprimulgiformes*** | *Caprimulgidae* | Nightjar | 1 |  |  | |  |  |
| ***CoraciiformesCoracii*** | *Coraciidae* | Roller | 5 |  |  | |  |  |
|  | *Meropidae* | Bee-eater | 22 |  |  | |  |  |
|  | *Alcedines* | Kingfisher | 4 |  |  | |  |  |
| ***Upupiformes*** | *Upupidae* | Hoopoe | 11 |  |  | |  |  |
| ***Passeriformes*** | *Hirundidae* | Swallow | 12 |  |  | |  |  |
|  |  | Swand martin | 2 |  |  | |  |  |
|  |  | Pale sand martin | 2 |  |  | |  |  |
|  | *Alaudidae* | Skylark | 4 |  |  | |  |  |
|  |  | Bimaculated lark | 6 |  |  | |  |  |
|  |  | Calandra lark | 4 |  |  | |  |  |
|  |  | Lesser short-toed lark | 6 |  |  | |  |  |
|  | *Sturnidae* | Starling | 18 |  |  | |  |  |
|  |  | Rose-coloured starling | 12 |  |  | |  |  |
|  |  | Common Mynah | 4 |  |  | |  |  |
|  | *Motacillidae* | Grey-headed wagtail | 36 |  |  | |  |  |
|  |  | Yellow-headed wagtail | 8 |  |  | |  |  |
|  |  | Masked wagtail | 2 |  |  | |  |  |
|  |  | Citrine wagtail | 1 |  |  | |  |  |
|  |  | Tawny pipit | 4 |  |  | |  |  |
|  |  | Tree pipit | 2 |  |  | |  |  |
|  |  | Rock pipit | 6 |  |  | |  |  |
|  | *Lanidae* | Long-tailed Shrike | 1 |  |  | |  |  |
|  |  | Isabelline shrike | 6 |  |  | |  |  |
|  | *Turdidae* | Bluethroat | 6 |  |  | |  |  |
|  |  | White-tailed rubythroat | 2 |  |  | |  |  |
|  |  | Wheatear | 34 |  |  | |  |  |
|  |  | Isabelline wheatear | 2 |  |  | |  |  |
|  |  | Nightingale | 6 |  |  | |  |  |
|  |  | Blackbird | 14 |  |  | |  |  |
|  |  | Rock thrush | 6 |  |  | |  |  |
|  |  | Blak-throated thrush | 4 |  |  | |  |  |
|  |  | Red-throated thrush | 1 |  |  | |  |  |
|  |  | Eversmann's redstart | 1 |  |  | |  |  |
|  | *Paridae* | Great tit | 3 |  |  | |  |  |
|  |  | Grey tit | 2 |  |  | |  |  |
|  | *Paradoxornitidae* | Bearded tit | 2 |  |  | |  |  |
|  | *Ploceidae* | Rock sparrow | 2 |  |  | |  |  |
|  |  | Spanish sparrow | 64 |  |  | |  |  |
|  |  | Indian sparrow | 23 |  |  | |  |  |
|  | *Fringillidae* | Chaffinch | 42 |  |  | |  |  |
|  |  | Brambling | 17 |  |  | |  |  |
|  |  | Linnet | 4 |  |  | |  |  |
|  |  | Common rosefinch | 6 |  |  | |  |  |
|  |  | Goldfinch | 2 |  |  | |  |  |
|  |  | Greenfinch | 2 |  |  | |  |  |
|  |  | Mongolian finch | 2 |  |  | |  |  |
|  | *Sylviidae* | Chiffchaff | 25 |  |  | |  |  |
|  |  | Paddyfield warbler | 2 |  |  | |  |  |
|  |  | Yellow-browed warbler | 4 |  |  | |  |  |
|  |  | Hume's warbler | 34 |  |  | |  |  |
|  |  | Greenish warbler | 2 |  |  | |  |  |
|  |  | Cetti's warbler | 4 |  |  | |  |  |
|  |  | Great Reed Warbler | 2 |  |  | |  |  |
|  |  | Blyth's warbler | 26 |  |  | |  |  |
|  |  | Glamorous Reed warbler | 12 |  |  | |  |  |
|  |  | Lesser whitethroat | 6 |  |  | |  |  |
|  | *Corvidae* | Rook | 150 |  |  | |  |  |
|  |  | Raven | 3 |  |  | |  |  |
|  |  | Desert raven | 13 |  |  | |  |  |
|  |  | Crow | 14 |  |  | |  |  |
|  |  | Hooded crow | 19 |  |  | |  |  |
|  |  | Jackdaw | 10 |  |  | |  |  |
|  |  | Magpie | 3 |  |  | |  |  |
|  | *Emberizidae* | Reed bunting | 4 |  |  | |  |  |
|  |  | Grey-necked bunting | 2 |  |  | |  |  |
|  |  | Red-headed bunting | 2 |  |  | |  |  |
|  |  | Pine bunting | 2 |  |  | |  |  |
|  |  | Corn bunting | 1 |  |  | |  |  |
|  |  | Yellowhammer | 8 |  |  | |  |  |
| ***Total 17*** | ***38*** | **183** | **6913** | **23** | **13** | | **1** | **8** |
